# An experimental loop design for the detection of constitutional chromosomal aberrations by array CGH

**DOI:** 10.1186/1471-2105-10-380

**Published:** 2009-11-19

**Authors:** Joke Allemeersch, Steven Van Vooren, Femke Hannes, Bart De Moor, Joris Robert Vermeesch, Yves Moreau

**Affiliations:** 1 MicroArray Facility, VIB, Leuven, Belgium; 2 ESAT-SISTA, KU Leuven, Leuven, Belgium; 3 Center for Human Genetics, University Hospital Gasthuisberg, Leuven, Belgium

## Abstract

**Background:**

Comparative genomic hybridization microarrays for the detection of constitutional chromosomal aberrations is the application of microarray technology coming fastest into routine clinical application. Through genotype-phenotype association, it is also an important technique towards the discovery of disease causing genes and genomewide functional annotation in human. When using a two-channel microarray of genomic DNA probes for array CGH, the basic setup consists in hybridizing a patient against a normal reference sample. Two major disadvantages of this setup are (1) the use of half of the resources to measure a (little informative) reference sample and (2) the possibility that deviating signals are caused by benign copy number variation in the "normal" reference instead of a patient aberration. Instead, we apply an experimental loop design that compares three patients in three hybridizations.

**Results:**

We develop and compare two statistical methods (linear models of log ratios and mixed models of absolute measurements). In an analysis of 27 patients seen at our genetics center, we observed that the linear models of the log ratios are advantageous over the mixed models of the absolute intensities.

**Conclusion:**

The loop design and the performance of the statistical analysis contribute to the quick adoption of array CGH as a routine diagnostic tool. They lower the detection limit of mosaicisms and improve the assignment of copy number variation for genetic association studies.

## Background

**Figure 1:**
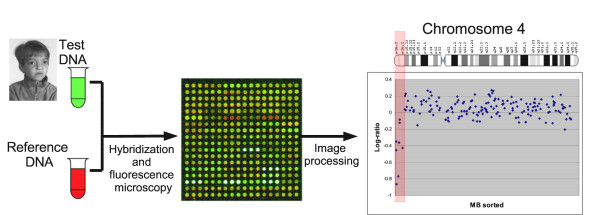
**BAC-based Array CGH**. Wolf-Hirschhorn syndrome was discovered in 1961 by Herbert Cooper and Kurt Hirschhorn. The phenotypical features include mental retardation, distinct facial appearance (typical Greek warrior helmet faces, high forehead), and seizures. Wolf-Hirschhorn is characterized by a deletion of the end of the short arm of chromosome 4; in particular, a deletion of the terminal band (4p16.3) is essential for full expression of the phenotype. Wolf-Hirschhorn can be detected with array CGH by comparing a genomic DNA sample of the patient (test) with that of a normal individual (reference). DNA extracted from test and reference sample is labeled with different fluorescent dyes (typically Cy3 and Cy5) and hybridized to the microarray. Array CGH probes can be PCR-amplified Bacterial Artificial Chromosomes or BAC clones or spotted long oligos. The microarray is scanned by two-channel laser scanner and aneuploid chromosomal regions are detected as probes with a deviant log ratio. This example clearly indicates a deviation of the log ratios at the end of the short arm of chromosome 4 and allows to confirm the hypothesis of Wolf-Hirschhorn syndrome.

Array Comparative Genomic Hybridization (array CGH) [1,2], also called molecular karyotyping [3], detects copy number aberrations and variations at high resolution on a genomewide scale [4,5]. Genomewide array CGH has been applied to detect chromosomal imbalances in patients with congenital anomalies and mental retardation [6-12]. An illustrative example is presented in Figure 1. Array CGH is a highly effective technique that is entering routine clinical use much faster than other microarray technologies. Indeed, compared to, for example, expression microarrays, array CGH enjoys several technical advantages: (1) genomic DNA samples are less prone to degradation than mRNA samples, (2) genomic DNA samples show much less variation between biological replicates than mRNA samples, and (3) interpretation of chromosomal imbalances is much easier than that of expression fingerprints. These advantages explain why array CGH for the diagnosis of constitutional anomalies is progressing faster towards the clinic than expression microarrays for the prediction of clinical outcome (e.g., in cancer), for which a few applications are now entering clinical practice [13-15].

Array CGH mostly competes with and is complementary to conventional karyotyping and Fluorescent *In Situ *Hybridization (FISH). Compared to conventional karyotyping, it offers a resolution between 10 kb and 1 Mb, instead of about 5 Mb, and detects at least twice as many aberrations [12]. Furthermore, it does not require the use of metaphase chromosomes, which makes it faster and less labor intensive. However, current array CGH techniques cannot detect balanced translocations, while this is straightforward with conventional karyotyping. Compared to FISH, array CGH provides genomewide coverage, instead of covering only a limited set of probes--so, it does not require prior knowledge of which aberration might be present (based on the phenotype of the patient).

The most frequent experimental setup for array CGH consists in comparing genomic DNA of a patient (test) with that of a normal individual (reference) using a two-channel microarray consisting of DNA segments spread across the whole genome. In the case of our clinical platform, the DNA segments consist in PCR-amplified BAC clones. However, the discussion applies equally to spotted long oligo platforms. So, we will refer to our probes as *reporters*. DNA from the test and reference samples is extracted, labeled with different fluorescent dyes (usually Cy3 and Cy5), hybridized to the microarray, and then scanned by two-channel laser scanner. Aneuploid chromosomal regions are detected as probes with a deviant log ratio of the intensities of the test against reference signal (approximately log_2_(1/2) for a deletion and log_2_(3/2) for a duplication). Usually the experiment is repeated in a dye-swap with the uorescent labeling of test and reference exchanged. The signals are then averaged over the dye-swap replicates to reduce the signal-to-noise ratio.

**Figure 2:**
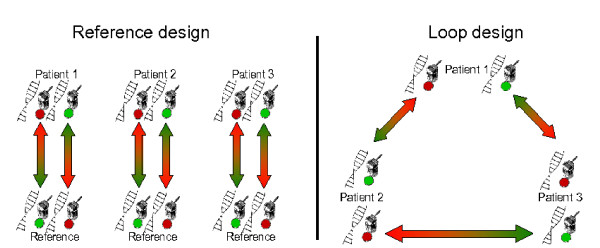
**The loop design**. Schematic overview of a reference and a loop design, in which three patients are compared. In the reference design, the three patients are compared to the DNA of a normal individual (reference). In the loop design the three patients are compared two-by-two.

An alternative design [10] is a loop design in which three hybridizations are carried out with three test patients that are compared with each other: Patient 1 versus Patient 2, Patient 2 versus Patient 3, and Patient 3 versus Patient 1, as shown in Figure 2. This design measures the intensities of three test samples in a statistically balanced way and requires no normal reference sample. Hence, only three arrays are used to analyze three patients and to obtain two measurements for each of them. For the classical dye-swap design, half of the resources are consumed to measure the reference sample of a normal individual and, therefore, six arrays would be necessary to obtain as many measurements from the test samples.

Extensive genomic variation (called copy number variation (CNV)) is also present in normal individuals [16-18]. The extent of this variation is surprising (covering at least 10% of the genome) and likely to have major implications for human variation and disease. In the classical dye-swap design, a deviant log ratio for one reporter in the test sample could just as well be associated with a variation in the reference sample. The difficulty in disambiguating deviations between the test and reference sample prevents us also from replacing the reference sample with a second test sample in the dye-swap design. The loop design, on the contrary, unambiguously associates a deviation to the correct sample by looking for a unique pattern of log ratios. For example, a duplication in Patient 1 will be associated approximately to a positive log ratio in the Patient 1 vs. Patient 2 hybridization, a negative log ratio in the Patient 3 vs. Patient 1 hybridization, and a null log ratio in the Patient 2 vs. Patient 3 hybridization. No deletion or duplication in another patient will display the same pattern, so the association is unambiguous. Another way to elevate this issue in dye swap experiments would be to use a DNA sample, pooled from several individuals. However, for frequently occurring CNVs, the intensity ratios will be reduced and, therefore, pooling will rather complicate data interpretation instead of simplifying [10].

For the statistical analysis, we consider two approaches for array CGH: linear modeling of the log ratios and mixed modeling of the absolute signal intensities. We compare both methods on a test data set consisting of 27 patients (9 loop designs) and we implement the method with the best signal-to-noise ratio as a user-friendly web application. Both methods analyze the data in a clone by clone way. On high resolution arrays, the resulting estimates can however be used as input for segment wise analysis techniques. Experimental designs that make the best use of available resources are essential for the widespread adoption of array CGH as a routine clinical tool for the diagnosis of constitutional chromosomal aberrations. Reduction of false positives and negatives guarantees the best service to the patient and the best use of economic resources, which are key factors in a clinical environment. Furthermore, correct assignment of "benign" copy number variations to the right sample will be important in upcoming studies of association between copy number variations and disease. Finally, this design will also lower the detection limit for mosaicism (i.e., chromosomal aberrations present in only a percentage of the cells).

Array CGH for the detection of congenital chromosomal aberrations is also a key method for the genomewide discovery of gene function. Patients with chromosomal aberrations provide a natural form of forward genetics screen. Through genotype-phenotype correlations, phenotypes can be associated to chromosomal regions containing only a few to a few tens of candidate genes [19]. Further prioritization of candidate genes using bioinformatics approaches and validation in small animal models (for example, zebrafish or fruit fly) allows rapid identification of disease causing genes [20].

## Results and Discussion

### New statistical models for the analysis of loop designs for array CGH

Two main philosophies vie for dominance for the statistical modeling of microarray signal intensities: (1) linear models of log ratios of intensities and (2) mixed models of absolute intensities. Two-channel microarrays were originally developed so that taking the ratio between the Cy3 and Cy5 intensity of a spot would eliminate multiple sources of variations (in particular, the amount of DNA material per spot). Linear models of log ratios keep in track with this philosophy and extend it by observing that log ratio measurements from different hybridizations containing equivalent samples are interdependent. They formulate these dependencies as a set of linear relations that are then inverted to obtain tighter estimates for a smaller set of independent statistical effects (which remain in essence similar to log ratios). By contrast, mixed models of absolute intensities aim at disentangling the signals from the Cy3 and Cy5 channels by expressing the intensities as the sum of an extensive set of fixed and random effects dependent on many factors that systematically affect the microarray measurements (dye effect, array effect, spot effect, etc.). Although the mixed models we consider are linear mixed models and thus also linear models, we will for simplicity refer in this paper to the two classes of methods as being "linear models" vs. "mixed models", respectively.

#### Mixed model of absolute signal intensities

We will apply a mixed model as proposed by [21]. These models were originally applied to cDNA microarrays, but they can be tailored to the analysis of array CGH. Before applying a mixed model, we want to correct hybridization signals for possible spatial effects (which cannot be easily corrected with the mixed model). We therefore apply a 2D spatial loess correction to the hybridization intensities and obtain loess corrected log ratios, from which we can derive corrected intensities. The mixed models proposed by [21] consist of two successive models: the normalization and the reporter-specific model. The normalization model corrects for array, dye, and patient effects. The fitted model can be written as *y*_*cij *_= *μ *+ *P*_*i *_+ *A*_*j *_+ PA_*ij *_+ *r*_*cij*_, where *y*_*cij *_are the Cy3 and Cy5 intensities for Clone *c*, Patient *i*, and Array *j*, *μ *is the overall average, *P*_*i *_is the fixed patient effect with three levels (*i *= Patient 1, 2, or 3), *A*_*j *_estimates the random array effect (also with three levels *j *= Array 1, 2, or 3), and PA_*ij *_fits the interaction effect between the patient and array effect, and in this way it also corrects for the dye effect. For each reporter, we extract the residuals *r*_*cij *_from the normalization model and fit a reporter-specific model *r*_*cij *_= *C*_*c *_+ CP_*ci *_+ CA_*cj *_+ *ε*_*cij*_, where *r*_*cij *_are the residuals obtained from the normalization model, *C*_*c *_is the overall average for Reporter or Clone *c*, CP_*ci *_is the fixed patient effect for Reporter *c *with three levels (*i *= Patient 1, 2, or, 3), CA_*cj *_estimates the random array effect for Reporter *c *(also with three levels *j *= Array 1, 2, or 3), and *ε*_*cij *_fits the random error effect. Our main interest is in the estimates of the CP_*ci *_effects, which reflect the difference between the patients for Reporter *c*. Specifically, we assess whether the contrasts Patient 2 vs. Patient 1 (= CP_*c*2 _- CP_*c*1_) and Patient 1 vs. Patient 3 (= CP_*c*1 _- CP_*c*3_) are equal to zero with a Wald's *F*-test. In the case where the contrast is significantly larger than zero for a chosen significance level *α*, we call this contrast *positive*. In the case where it is smaller than zero, it is called *negative*. Else we assign 0. Based on both hypothesis tests, the reporters are classified as duplicated or deleted according to the classification shown in Table 1. For example, if the contrast Patient 1 vs. Patient 3 (= CP_*c*1_- CP_*c*3_) is positive and the contrast Patient 2 vs. Patient 1 (= CP_*c*2_- CP_*c*1_) is negative for a reporter, then this reporter is likely to be duplicated for Patient 1. In some rare cases, we obtain as result a reporter that has, for example, a negative value for both contrasts CP_*c*1 _CP_*c*3 _and CP_*c*2 _CP_*c*1_, which is none of the combinations in Table 1. In this case, we call the reporter *strange*.

**Table 1:** Classification of the reporters.

Classification	Log Ratio patient_1_/patient_3_	Log Ratio Patient_2_/patient_1_
Duplication for patient_1_	positive	negative

Duplication for patient_2_	0	positive

Duplication for patient_3_	negative	0

Deletion for patient_1_	negative	positive

Deletion for patient_2_	0	negative

Deletion for patient_3_	positive	0

Each reporter is classified as duplicated (*positive*), deleted (*negative*), or not changed in copy-number (0) for two chosen contrasts, i.e. Patient 1 versus 3 and Patient 2 versus 1. Based on the results of both contrasts a reporter can be recognized as duplicated or deleted for Patient 1, 2, or 3, according to this scheme.

#### Linear model of log ratios

An alternative statistical tool is a linear model of the log ratios, as proposed by [22]. In contrast to the mixed model, this technique fits the 2D spatial loess corrected log_2 _ratios directly. In this particular case, we can choose the following contrasts C_*c*1 _= log_2_(*P*_*c*1_/*P*_*c*3_) and C_*c*2 _= log_2_(*P*_*c*2_/*P*_*c*1_), where *P*_*ci *_corresponds to the true underlying signal for Reporter *c *for Patient *i*. These contrasts correspond to the samples that were directly compared on the first two slides in Figure 2 and the observed log ratios on these slides should on average be equal to the contrast. The data of the third slide should then correspond on average to C_*c*3 _= log_2_(*P*_*c*3_/*P*_*c*2_) = *C*_*c*1_-*C*_*c*2_. The linear model that fits the data can be written as

where *E *denotes the expectation of a random variable, *X *is the matrix of linear dependencies, *C*_*c *_is the vector of contrasts for Reporter *c*, and *y*_*ci *_denotes the log_2 _ratio for Reporter *c *measured on the *i*^*th *^slide. For each reporter, the least squares estimates of the three contrasts are obtained. To classify the contrasts as significantly duplicated, deleted, or not changed in copy-number, we apply the moderated *t*-statistic as implemented in the Bioconductor package limma, which implements linear models for microarray data analysis [22]. The *p*-values from the moderated *t*-test were corrected to control the false discovery rate with Benjamini-Hochberg [23]. Similarly to the mixed model, we can detect reporters that are duplicated or deleted for a patient, based on the *p*-values of the contrasts. For a chosen cut-off value *α*, we decide whether a reporter is not differentially expressed (0), upregulated (*positive*), or downregulated (*negative*) for a contrast. Based on the two contrasts, we can again classify a reporter as duplicated or deleted for a patient according to Table 1. Again, we can on rare occasions obtain *strange *reporters.

### Model validation

The mixed model and linear model provide two distinct ways to analyze the loop design experiments. To decide which method is preferable, we will first check which estimation method best separates the aberrant from the non-aberrant reporters. This will already give an indication to which method is preferred. Secondly, we will compare the false positive and false negative rates for a number of cut-off values *α*. Based on this information, we will decide which method to use and choose a cut-off value to call a reporter significantly duplicated or deleted. For the comparison of the analysis approaches, we consider a data set consisting out of nine loop designs from patients seen at our genetics center (Center for Human Genetics, U.Z.Leuven; see Table 2 and Methods section). Fifteen of the 27 patients involved in the nine loop designs showed one or more confirmed deletions or duplications of a chromosomal segment. Two experiments (Experiments 1 and 9) in our test data set include a sex mismatch. As for these experiments, the Y chromosome is absent for at least one of the patients, the measurements on the Y chromosome were excluded for both experiments from all computations. Because the X chromosome has regions with chromosome Y homology, the intensity ratios of chromosome X reporters are also more variable, and hence the X chromosome was also excluded from the computations for Experiments 1 and 9. For each of the nine loop designs, we classify the reporter as being deleted/duplicated if the reporter was classified as deleted or duplicated for one of the patients in the loop design. If the reporter was not deleted/duplicated for any of the patients in the loop design, the reporter was classified as non-aberrant. In total, this data set comprises 328 aberrant reporters: 116 deleted and 212 duplicated reporters. Over all nine experiments, we have a set of 30,668 measurements for non-aberrant reporters for any of the three patients in the loop.

**Table 2:** Loop design test data set.

Experiment	Patient	Deletions/duplications	length	Confirmed with
1	1	deletion on 13	25	karyotyping
	2	duplication of X (sex mismatch)	149	karyotyping

2	1	duplication of 18	102	karyotyping and FISH

3	1	deletion on 10	6	
	2	duplication on 7	20	
	3	duplication on 15	43	

4	2	deletion on 4	15	FISH
	3	Inversion (p11-q13)		karyotyping

5	1	deletion on 12	14	FISH

6	1	deletion on 9	6	
	3	deletion on 12	7	

7	2	duplication on 5	13	
	2	deletion on 18	16	

8	1	duplication on 13	15	FISH
	1	deletion on 13	12	FISH
	2	balanced translocation (2;6)(q33,1;p23)		karyotyping

9	1	duplication on 7	11	
	1	deletion on 7	15	
	2	deletion of X (sex mismatch)	158	karyotyping
	3	duplication on 21	8	

The nine loop design experiments, listed in the table, will be used as a test data set to compare the two methods, linear model and mixed model. For each aberration present in the experiment the number of deleted or duplicated reporters is indicated.

#### Signal-to-noise ratios

Assessing which method is best capable of distinguishing between the intensities of aberrant and non-aberrant reporters can be done by computing *signal-to-noise-ratios *(SN) (for both deletions and duplications separately) as

where alteration type is deletion or duplication. As we have collected a data set with 212 duplicated reporters, 116 deleted, and 30,668 non-aberrant reporters, we can compute the SN values based on the absolute values of the contrasts Patient 1 vs. Patient 3 and Patient 2 vs. Patient 1, for both the linear model and the mixed model. The results are shown in Table 3. As for deleted clones the difference between their log ratios (± ) and log ratios of non-aberrant clones is larger than for the log ratios derived from duplicated clones (± ), the signal-to-noise-ratio is of course larger for the deleted reporters than for the duplicated reporters. The linear model leads to a significant reduction in the noise, especially for the non-aberrant reporters, and this results in a larger signal-to-noise-ratio. Therefore, these statistics are favorable to the linear model.

**Table 3:** Signal-to-noise-ratios.

	Mixed model	Linear model
mean_non-aberrant_	0.00701	-0.00064
s.d._non-aberrant_	0.11306	0.06914

mean_dupl_.	0.48515	0.48777
s.d._dupl_	0.12920	0.11967
**SN_dupl_**	**3.93861**	**4.99782**

mean_del_	0.76779	0.78468
s.d._del_	0.22275	0.18787
**SN_del_**	**4.30702**	**5.54778**

For both methods (i.e., the mixed model and the linear model), the number of reporters, average and standard deviation of the average log_2_-ratios are given for the non-aberrant (# = 30,668), duplicated (# = 212), and deleted reporters (# = 116). Based on these numbers the signal-to-noise-ratios are computed

#### True positive and false positive rate

**Figure 3:**
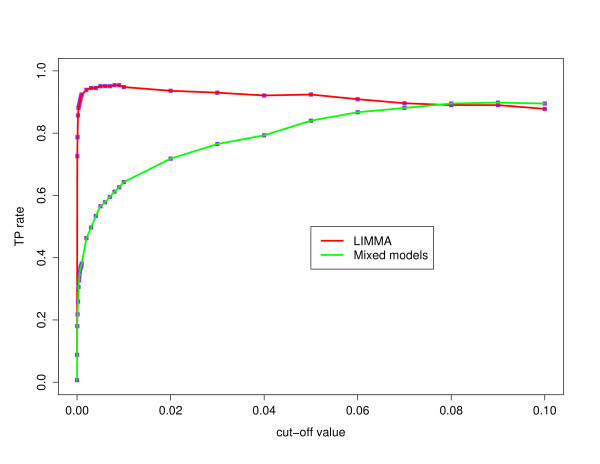
**True positive rate**. For a range of significance levels *α*, we compute the true positives rate (TP) for both the mixed model and the linear model. These true positive (TP) rates (*y*-axis) are plotted against the significance levels *α *(*x*-axis) and connected with a green line for the mixed model and a red line for the linear model.

**Figure 4:**
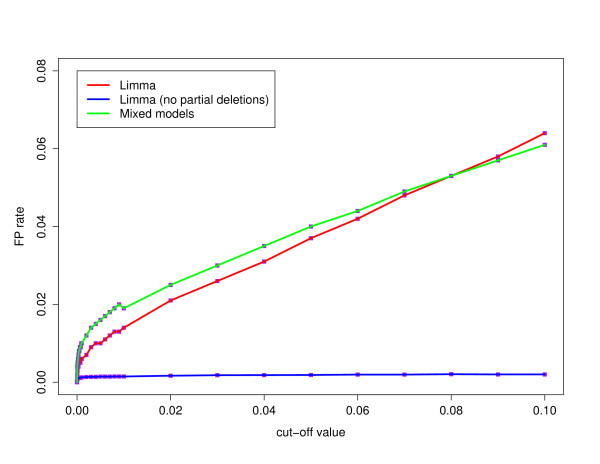
**False positive rate**. For the mixed model and the linear model, the false positive (FP) rates are plotted for the range of significance levels *α *with a green and red line, respectively. The FP rate for the complete deletions or duplications, obtained via the linear model is indicated in blue.

First, we compute for a number of significance levels *α*, the percentage of the duplicated and deleted reporters that are correctly classified as duplicated and deleted, respectively, in our test data set, according to both methods. The true positive (TP) rates are shown for the mixed model and the linear model in Figure 3 in function of the significance level *α*. For the linear model, the TP rate reaches a maximum of 0.954 for significance level *α *= 0.009. For higher *α*, surprisingly, the TP rate drops a little. This effect results from the fact that some reporters become *strange *reporters for larger significance levels *α*. For the classification with the mixed model, the TP rates grow slowly as the significance level increases. Within this range of significance levels *α*, it never reaches the maximum TP rate value obtained with the linear model. Perhaps it comes closer to the result obtained with the linear model if we allow for even larger significance levels *α*, but this will increase the FP rate and the number of strange reporters to an unacceptable level. Outside the duplicated and deleted regions, other reporters were also classified as duplicated or deleted. These positives can be false positives, due to technical artifacts, or they can indicate true biological variations. At this point, we will not make the distinction between both kinds of aberrant reporters, as it does not affect the method comparison, and we will refer to this set of positives as non-confirmed positives. At a later stage, this set of non-confirmed positives will be examined in depth, for one method and one significance level *α*. The number of these non-confirmed positives is shown in Figure 4. The figure shows that there is no clear difference in the non-confirmed positive rates between both methods. For low significance levels *α *the linear model has a slightly smaller number of non-confirmed positives. The combined results on the signal-to-noise ratio, the TP, and non-confirmed positive rate lead to the conclusion that the linear model is the preferable method.

### Optimization of the linear model

In the previous section, we focused on how well the different methods fit the measurements by assessing their capabilities to divide the non-aberrant reporters from the deviating reporters and by comparing the FP and TP rates. This indicated that the linear model was best suited to distinguish these groups of reporters, although also the linear model has a fairly high FP rate. However, we did not yet benefit from all available information.

#### Completely and partially deleted reporters

To further optimize the reporter classification, we will use the fact that if, for example, a reporter of Patient 1 is deleted or duplicated, its contrast Patient 2 vs. Patient 1 should theoretically be equal to log_2 _() = 1 or log_2 _() = -0.58, respectively. However, nonlinear saturation effects in the signals cause a deviation from these values. Instead of taking the theoretically expected values (i.e., ± 1 and ± 0.58), we will estimate the expected values based on the linear model estimates of the contrasts for the group of confirmed deletions and duplications, after exclusion of the deletions and duplications on the X and Y chromosome. This results in an average for the absolute log ratios of 0.86 for the deleted reporters and 0.54 for the duplicated reporters. Therefore, if we detect with the linear model a reporter that is likely to be duplicated or deleted, we extract its contrasts *C*_*c*1 _and *C*_*c*2_. If their absolute value is not larger than 0.54 or 0.86, respectively, we use an adapted version of the moderated *t*-test, as implemented in the limma package. We use the same standard deviation of the contrasts, as computed within the previous limma procedure, and test one-sidedly the hypothesis *H*_0 _: |*C| *= 0.54 versus *H*_*a *_: |*C*| < 0.54 for the duplicated reporters and *H*_0 _: |*C| *= 0.86 versus *H*_*a *_: |*C*| < 0.86 for the deleted reporters. If we cannot reject the hypothesis at a significance level *α*_partial _for both tests, we call the reporter *completely *deleted or duplicated, else we call the reporter *partially *deviating (i.e., only a part of the clone has been deleted/duplicated instead of the complete clone). As a significance level, we choose *α*_partial _= 0.01. For this significance level, the non-confirmed positives restricted to the reporters that are completely deleted are plotted as a blue line in Figure 4. This non-confirmed positives rate ranges between 0.001 and 0.002.

#### The non-confirmed positives

The non-confirmed positives rate obtained in the previous section is not a direct indication of the false positives rate, as they can comprise not only false positives, but also both true positives or polymorphic reporters. In our data set of nine loops, we saw that after extraction of clones that were proven to be copy number variable regions, 15 single clones with complete deletions or duplications are picked up by our method, at a significance level of *α *= 0.001. Seven of these clones have been previously described as polymorphic [24]. One clone (RP1-93N13) overlaps with a CNV present in the normal population (Genomic Database of Variants; http://projects.tcag.ca/variation/). To determine whether the remaining reporters are false positives or true CNVs, qPCR or FISH [10] was performed. For six clones, the deletion or duplication was confirmed. For one clone we could not determine the copy number variation due to lack of DNA. The remaining four clones were all measured in Experiment 1 and caused by low spot quality, as they had a large discrepancy between the mean and the median pixel intensities and a large standard deviation of the pixel intensity. Hence, our method reliably detects aberrant clones. An overview of these results is shown in Table 4.

**Table 4:** The non-confirmed positives.

Clone	Status	Validation method
*RP11-114K7*	[24]	

*RP11-161M6*	[24]	

*RP1-225D2*	[24]	

*RP6-14C6*	[24]	

*RP11-342F21*	[24]	

*RP11-342F21*	[24]	

*RP6-22D12*	[24]	

*RP1-93N13*	Genomic Database of Variants	

*RP11-2P5*	Single clone deletion	qPCR

*RP11-48E16*	Single clone deletion	qPCR

*RP11-127A9*	Single clone deletion	qPCR

*RP11-469N6*	Single clone deletion	qPCR

*RP11-576I16*	Single clone duplication	qPCR

*RP11-152O18*	Single clone deletion	FISH

*RP11-361M10*	Not confirmed	Lack of DNA

*RP11-404P12*	Low spot quality	

*RP5-982E9*	Low spot quality	

*RP11-301H15*	Low spot quality	

*RP4-742J24*	Low spot quality	

Outside the duplicated and deleted regions, 15 reporters were also classified as being completely duplicated or deleted. Eight of them could be confirmed as being polymorphic clones according to [24] and the Genomic Database of Variants. Six of them confirmed with FISH or qPCR. One clone could not be confirmed, due to lack of DNA.

At *α *= 0.001, also 13 clones were classified as being *strange*. Ten of these clones overlapped with CNVs that are present in the normal population, according to the Genomic Database of Variants. (Note also that not all normal CNVs have already been identified.) Therefore, we suspect that a *strange *clone can often be explained as a polymorphic clone that is shared by two patients. Taken together these results indicate that at *α *= 0.001 our procedure has both low false negatives (TP rate around 95%) and essentially no false positives for completely deleted or duplicated clones. Most clones detected as positive or strange outside the regions known in the benchmark have been confirmed by qPCR or FISH; several of them being known normal CNVs.

### Web application

The method is implemented as a web application and is available at http://www.esat.kuleuven.be/loop. A demo account with test data is available. Currently, the application and the statistical analysis have been tested and refined on an in-house series of over 400 patients. Details on the implementation and the use of the web application can be found in Additional file 1.

## Conclusion

A first point of discussion is to which platforms the proposed statistical models are applicable. Some array CGH platforms are available using single-channel microarrays. In this situation, the loop design is simply not applicable. The loop design is directly dependent on using a two-channel microarray.

A second point of discussion is a main assumption that underlies our analysis, that two patients never share the same aberration. If two patients have an aberration for the same reporter, the statistical model cannot correctly interpret the result|resulting mostly into a *strange *reporter. For the detection of congenital anomalies, this effect is prevented by putting into a loop design patients who have clearly distinct phenotypic patterns. The rare cases where a common aberration is still present can be rescued at the validation stage. Incorrect assignment to the third patient would not be validated by FISH or qPCR and, in this case, the possibility of a common aberration for the two other patients should be kept in mind. Reporters flagged as *strange *can be validated in all three patients to clear out the situation.

At our genetics center, the pick-up rate (i.e., the percentage of patients that show an chromosomal aberration) is around 20%. Hence, a loop design consisting out of three patients with a CNVs occurs only in one out of 125 cases. The likelihood of having patients that share an abnormality is even smaller. However, this assumption is unrealistic when studying tumors or when doing preimplantation diagnosis. In the case of tumors, many chromosomal regions can be affected and overlap between patients is essentially unavoidable. In the case of preimplantation diagnosis, we have developed a procedure where one of the eight blastomere cells of an 8-cell embryo from *in vitro *fertilization is assessed by single-cell array CGH [25]. In this procedure it is essentially impossible to guarantee that the aberrations from different blastomeres do not overlap (for example, some aberrant embryos have chaotic genomes where many chromosomes are affected).

In some rare cases, deviating reporters cannot be assigned as duplicated or deleted, but are instead labeled as *strange*. We observed that the majority of these *strange *reporters corresponded to polymorphic reporters. This can correspond to the situation where two patients share a complex polymorphic reporter [16] and will be investigated more deeply in a subsequent study.

Our analysis was characterized by the fact that for our optimized threshold we have a low number of false negatives and close to no false positives. Given that a secondary validation is available through FISH, which can catch false positives but not false negatives, we would want to increase our threshold further to decrease the number of false negatives at the cost of a few false positives that can then be caught by the FISH validation. However, increasing the threshold further results rather in more reporters being labeled as *strange*, which we prefer to avoid.

At a significance level of 0.001, for 0.251% of the clones the intensity ratios were suggestive of partial deletions of duplications. Most likely, the intermediate intensity ratios reflect the presence of subclonal CNVs (i.e., only a part of the clone has been deleted/duplicated instead of the complete clone). Recently, several articles show high levels of small CNVs in the human genome [16-18,26]. Moreover, we performed qPCRs on some of these clones using several PCR primers and could confirm such partial deletions (data not shown). However, since no systematic analysis of all these clones has been performed, we do not know the number of false positives.

In our specific setting and in our hands, linear models clearly outperformed mixed models. We do not draw any conclusion with regard to the suitability of one class of method versus the other in general. In our setting, we hypothesize that the mixed model was less robust to deviation from the underlying normality assumptions or that the more compact (fewer parameters) estimation procedure of the linear model increased its robustness. The improvement of the mixed model in this setting appears to be an interesting research direction.

Microarray CGH for the diagnosis of congenital chromosomal aberrations is progressing rapidly from the research lab to the clinic. What value do such improved statistical procedures add to the diagnosis? First of all, increasing the signal-to-noise ratio between aberrant and normal clones through our statistical procedure is likely to improve the detection of low-grade mosaicism (i.e., chromosomal aberrations present in only a percentage of all cells), which is currently difficult to detect by array CGH. In a previous study [25], we presented a power analysis model for the detection of low-grade mosaicism where the signal-to-noise ratio was a critical factor in determining the limit of detection for mosaicism.

Secondly, CNVs are hard to assign with certainty to the patient instead of the "normal" reference. While these CNVs are currently largely handled as noise, some of them are likely to act as genetic modifiers that are risk factors for disease or modulate its penetrance and phenotypic spectrum [16]. Association studies are currently being started to evaluate the importance of such CNVs in both congenital and acquired disorders. Unambiguous assignment of the "normal" CNVs to the correct sample is therefore paramount. And as a last point, there is the issue of cost per experiment. To keep cost down, some laboratories do not perform a dye swap when using the basic array CGH setup (patient vs. normal reference). In this case, a single array is used per patient. However, only one measurement per patient will be available in this case instead of two with our design, which means a higher level of false positives and negatives. False positives will be caught by the secondary FISH or qPCR validation, but false negatives will not. This results in lower quality diagnostics for the patient.

In conclusion, our results indicate that the experimental loop design, together with a statistical analysis by a linear model, provides an efficient procedure for the detection of chromosomal aberrations in congenital anomalies by array CGH. It is significantly superior to the classical setup by doubling the use of resources and unambiguously assigning variation to the correct patient. These improvements have a direct impact on the diagnosis offered to the patient using the microarray technology that is closest to routine clinical use.

## Methods

### Array CGH

A 1 Mb resolution BAC array was performed as described in [10]. In short, 3500 BAC en PAC clones from the Welcome Trust Sanger Institute were amplified by two rounds of DOP-PCR [27]. The purified aminolinked PCR products were spotted in duplicate on 3-D CodeLink Bioarray System slides (Amersham Biosciences, Piscataway, NJ). 150 ng of patient DNA was labeled by random prime labeling system (BioPrime Array CGH Genomic Labeling System, Invitrogen) using Cy3- and Cy5-dCTPs (Amersham Biosciences). Probe concentration and labeling efficiencies were measured using the nanodrop ND-1000 spectrophotometer (Nanodrop Technologies, Rockland, DE). The probe was placed on the slide under a glass cover slip (24 × 24 mm). The slides were incubated for 42-72 hours at 37°C under humified conditions.

Post hybridization washing was performed by soaking in 1 × PBS for 10 minutes and followed by a stringency wash on 42°C for 30 minutes in 50% formamide 2× SSC solution and 10 minutes in 1× PBS at room temperature. To finalise, the slides were dried by centrifugation for at least 1 minute.

### Image and data analysis

After the washing, a two-channel scan was performed with an axon laser scanner GenePix 4000B (Molecular Devices, Union City, CA) at 532 nm and 635 nm using GenePixPro 6.0 program. The results are converted into GPR format and can be directly uploaded to the web-based application. The median spot intensities were corrected with the local median background, and only those spots with a signal above background (i.e., foreground intensity larger than local background intensity plus twice the local standard deviation of the background) were retained for the analysis. In this way, only few spots are lost, as almost all spots are above background (on average 96.6%). The ratios of the Cy5 to the Cy3 intensities were computed for each reporter and base 2 log transformed. The log ratios are normalized using a 2D spatial loess normalization, in which one applies a loess regression to fit the log_2_-ratios (*M*-values) on the coordinates on the slide as predictor variables.

### Benchmark data set

For the comparison of the analysis approaches, we consider a data set consisting out of nine loop designs or 27 patients with mental retardation (MR) and multiple congenital anomalies (MCA). The patients were seen at our genetics center (Center for Human Genetics, U.Z.Leuven). Conventional karyotyping showed chromosomal imbalances in 11 patients. Analysis of the patients was carried out with a 1 Mb BAC array. In first instance, the data analysis strategy that we had previously developed was applied to this data. In this procedure, a region is called aberrant, if one clone passes the threshold of 4 × *SD *and if two or more flanking clones were passing the threshold of log_2 _() - 2 × *SD *as described in [24]. If a deletion or a duplication larger then 3 Mb was detected, FISH was performed to confirm the results of the array. In case of a duplication smaller than 3 Mb, we performed quantitative PCR (qPCR) [10]. 16 out of 27 patients show one or multiple clone anomalies, whereas 10 patients are apparently normal, at least according to the results of the array. One of the patients was a carrier of an inversion and another patient had a balanced translocation. Both aberrations cannot be detected by array CGH, but with conventional karyotyping; as such these patients did not contribute data to the benchmark. A short summary of the data set is shown in Table 2. In total, this data set comprises 635 aberrant clones: 274 deleted and 361 duplicated clones.

The study was approved by the institutional review board and appropriate informed consent was obtained from human subjects. The data set has been uploaded to the Gene Expression Omnibus (Accession number GSE6538) and is publicly available.

## Consent

Written informed consent was obtained from the patient for publication of accompanying images. A copy of the written consent is available for review by the Editor-in-Chief of this journal.

## Authors' contributions

YM, JV, and BDM coordinated the study and formulated the methodology and research schema. JA performed the statistical analysis. JA and SVV contributed to the implementation. FH carried out the microarray experiments and did the validation experiments. JA, FH, YM, and JV interpreted the results. JA and YM wrote the manuscripts. All authors read and approved the final manuscript.

## Supplementary Material

Additional file 1 **Documentation on the web application**. This additional file provides a description of the web application, implementing the algorithm presented in this paper. Click here for file
