# Supplementary material for: An experimental loop design for the detection of constitutional chromosomal aberrations by array CGH
Source: BMC Bioinformatics. 2009 Nov 19;10:380. doi: 10.1186/1471-2105-10-380 (PMC2791104; doi:10.1186/1471-2105-10-380)
Supplement: Additional file 1 — Documentation on the web application. This additional file provides a description of the web application, implementing the algorithm presented in this paper. [file 1471-2105-10-380-S1.PDF]

Appendices

Documentation on the web application

The method is implemented as a web application and is available at www.esat.kuleuven.be/loop. A demo account with test data is available (http://www.microarrays.be/LoopTool.htm). Currently, the application and the statistical analysis have been tested and refined on an in-house series of over 400 patients.

Data processing

The application offers three components: an upload wizard for GPR files, a slide view that provides an overview of all uploaded hybridizations, and a loop design view offering reports of all loop design experiments.

The upload wizard expects the GenePix GPR file format. Other scanner manufacturers have included the possibility to export in this format in their scanner software. The upload wizard expects that unique identifiers be provided for Cy5 and Cy3 samples: these are used to verify loop designs as valid, and are used as sample references throughout the tool. The slide view provides an overview of basic information on uploaded hybridizations. Each hybridization record can be folded open to view technical information, and timing and lab information can be edited. In this view, three hybridizations that make up a single loop design are checked, and selected experiments are combined into a loop design. After submitting three hybridizations as a new loop design, a new entry is added in the loop design view. A status comment indicates what phase of the analysis is active. When done, four reports can be viewed: an overview, and three individual patient reports. The overview report will display experiment design and quality assessment information, Cy5 and Cy3 slide background images, and MA-plots before and after 2D loess normalization. Significantly aberrant reporters are shown in an overview, highlighting reporters previously marked as polymorphic. Potentially polymorphic reporters are shown in Table S1. If needed, a list of significantly aberrant reporters and a list of all reporters can be downloaded from this report as a tab delimited text file for further processing. A graphical overview shows normal and aberrant reporters ordered by chromosomal position for all hybridizations. The individual patient reports show quality statistics and a graphical display of significantly aberrant reporters. On this overview, the user can zoom in to individual chromosomes. A table provides an overview of aberrant reporters, which can again be downloaded as a tab delimited file.

Architecture

The web application consists of a set of Java Server Pages (JSP) that are run in Apache Tomcat Java application servers on two different machines. A single MySQL database instance with daily back up is used as a back-end data store and holds user and hybridization loop design data. GPR files are stored on RAID disks with daily backup, as are images and statistics results. Statistics and visualization scripts were written in R and make use of BioConductor modules. Instances of RServe, a daemon interface to R that accepts and handles remote calls from Java, are installed on two Unix machines for increased availability.

Table S1 - The list of potential polymorphic reporters.

Next to their identifier, an indication of the number of times it was detected as deviating, along with its chromosome (if determined).

Reporter ID	# detected	Chromosome
SC1_1Mb_PACE9	9	1
NONSC1B4	9	15
SC9BACMbset_1F12	7	17
NONSC24B9	7	5
SC10BAC-1Mbset-1E12	6	10
NONSC16A2	5	5
NONSC43G1	5	17
NONSC24A3	5	-
NONSC34A4	3	16
NONSC33A5	3	19
NONSC32E3	5	16
SC10BAC-1Mbset-1A3	4	10
SC6PAC1Mbset1F9	4	6
NONSC31C3	4	17
Cancer_1G8	3	5
SC13_1Mb_BAC1E4	3	13
SC13_1Mb_BAC1A5	4	Y
NONSC10B6	2	5
SC1_1Mb_BAC1G5	3	1
NONSC32B9	2	15
SC22_0.75_BACB8	2	22
NONSC8F4	3	17
NONSC10C7	3	7
NONSC2C8	2	8

Continued on next page

Continued from previous page

Reporter ID	# detected	Chromosome
NONSC3C7	2	2
NONSC8D3	2	2
telomereB8	2	14
telomereG1	2	2
NONSC40A4	2	8
NONSC40D1	2	4
NONSC41E2	2	8
NONSC11G2	2	8
NONSC10G9	3	2
NONSC10C3	2	16
Cancer_1A10	3	11
NONSC40E7	3	16
